# Effects of eptinezumab on self-reported work productivity in adults with migraine and prior preventive treatment failure in the randomized, double-blind, placebo-controlled DELIVER study

**DOI:** 10.1186/s10194-022-01521-w

**Published:** 2022-12-02

**Authors:** Piero Barbanti, Peter J. Goadsby, Giorgio Lambru, Anders Ettrup, Cecilie Laurberg Christoffersen, Mette Krog Josiassen, Ravinder Phul, Bjørn Sperling

**Affiliations:** 1 Headache and Pain Unit, IRCCS San Raffaele Pisana, Via della Pisana 235, 00163 Rome, Italy; 2 San Raffaele University, Rome, Italy; 3 NIHR King’s Clinical Research Facility, & Headache Group, King’s College London, London, UK; 4 The Headache Service, Guy’s and St Thomas’ Hospitals NHS Trust, London, UK; 5 H. Lundbeck A/S, Copenhagen, Denmark

## Abstract

**Background:**

The multinational phase 3b DELIVER trial was designed to evaluate the efficacy and safety of eptinezumab for migraine prevention in patients with prior preventive treatment failures across 17 countries. In the placebo-controlled portion, eptinezumab relative to placebo demonstrated greater reductions in migraine and headache frequency, migraine and headache severity, and acute medication use. The objective of this report is to describe the effects of eptinezumab on self-reported work productivity in the placebo-controlled portion of DELIVER.

**Methods:**

Adults 18–75 years of age with migraine and documented evidence of 2 to 4 prior preventive treatment failures in the past 10 years were randomized to receive eptinezumab 100 mg, 300 mg, or placebo intravenously (IV) every 12 weeks. The Work Productivity and Activity Impairment questionnaire specific to migraine (WPAI:M), which comprises 6 items (4 of which are completed by currently employed patients only), was administered every 4 weeks. Changes from baseline in subscores (absenteeism, presenteeism, work productivity loss, and activity impairment) were calculated based on item responses. A mixed model for repeated measures was used to analyze changes from baseline in WPAI:M subscores.

**Results:**

A total of 890 adults (mean age, 43.8 years) were included in the full analysis set (eptinezumab 100 mg, *n* = 299; eptinezumab 300 mg, *n* = 293; placebo, *n* = 298). Mean WPAI:M subscores at baseline indicated a negative impact of migraine attacks on work productivity and ability to complete normal daily activities. Eptinezumab improved WPAI:M subscores more than placebo at all assessment points throughout the study. Mean changes from baseline in self-reported work productivity loss were −19.5, −24.0, and −9.7 at Week 12; and −22.6, −20.2, and −7.2 at Week 24 (all *P* < 0.001 vs placebo) for eptinezumab 100 mg, eptinezumab 300 mg, and placebo, respectively. Mean changes from baseline in activity impairment were −21.3, −23.8, and −11.2 at Week 12; and −24.7, −22.6, and −10.1 at Week 24 (all *P* < 0.0001 vs placebo). Similarly, mean improvements in absenteeism and presenteeism were greater in the eptinezumab groups than in the groups receiving placebo at all timepoints (*P* < 0.05).

**Conclusion:**

In adults with migraine and prior preventive treatment failure, eptinezumab 100 mg and 300 mg IV every 12 weeks improved absenteeism, presenteeism, work productivity loss, and activity impairment more than placebo.

**Trial registration:**

ClinicalTrials.gov (Identifier: NCT04418765); EudraCT (Identifier: 2019–004497-25) (https://www.clinicaltrialsregister.eu/ctr-search/trial/2019-004497-25/PL).

**Graphical Abstract:**

Eptinezumab improves self-reported work productivity in patients with migraine and prior preventive treatment failures.

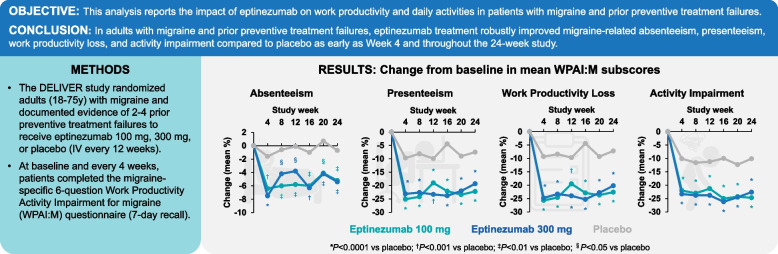

**Supplementary Information:**

The online version contains supplementary material available at 10.1186/s10194-022-01521-w.

## Introduction

Migraine is a disabling neurologic disorder that has not only been associated with poor work attendance (absenteeism), but also with reduced work effectiveness/work productivity while being at work (presenteeism) [1–4]. In fact, it has been estimated that patients with migraine lose between 3.2 and 89.2 work-equivalent days per year (average, 10.2 days), primarily due to presenteeism [5]. Annual economic losses due to presenteeism have been estimated to exceed $21 billion, and those due to absenteeism to be $2.7 billion [6]. As would be expected, reductions in productivity increase with increasing headache frequency [7], and work productivity loss commonly persists despite acute and/or preventive treatment [1, 2, 8]. If readily available, new migraine-specific treatments have the potential to reduce this financial burden by improving work productivity.

Eptinezumab (Lundbeck Seattle BioPharmaceuticals, Inc., Bothell, WA, USA) is a humanized monoclonal antibody that selectively binds to and inhibits the activity of calcitonin gene-related peptide (CGRP) [9]. It has demonstrated early onset of preventive effects as soon as day 1 after IV administration, as well as sustained migraine preventive effects and reduction of migraine burden with quarterly infusions over 2 years of continued treatment [10, 11]. Furthermore, eptinezumab demonstrates an acceptable safety/tolerability profile [10, 12–15] and reductions in disease impact are sustained with 2 years of continued treatment [16]. These characteristics have ultimately contributed to reduced disability, as well as to improved functioning and improved health-related quality of life.

The phase 3b DELIVER trial was designed to evaluate the efficacy and safety of eptinezumab for migraine prevention in patients with 2 to 4 prior preventive treatment failures. Many in the patient population of DELIVER were individuals who were eligible for reimbursement for anti-CGRP therapies [17]. The placebo-controlled portion of DELIVER demonstrated greater reductions in migraine and headache frequency, migraine and headache severity, and acute medication use with eptinezumab relative to placebo [17]. The objective of this report is to describe the effects of eptinezumab on patient-reported measures of work productivity in the placebo-controlled portion of the DELIVER trial.

## Methods

### Study design

The DELIVER study was conducted at 96 study locations in 17 countries (in the United States and Europe). The placebo-controlled period of the study was completed in October 2021, and the primary results have been published [17]. A detailed description of the study methodology was included in the initial report. Briefly, DELIVER is a phase 3b, multicenter, randomized, double-blind, placebo-controlled, parallel-group trial. Following a 28–30-day screening period, eligible patients participated in a 24-week placebo-controlled period and a 48-week dose-blinded extension period. The study was conducted in accordance with standards of Good Clinical Practice as defined by the International Conference on Harmonisation [18], as well as all applicable federal and local regulations. The local review board or central institutional review board or ethics committee approved the study at each site, and patients provided written informed consent prior to any study procedures. The DELIVER study is registered on ClinicalTrials.gov (NCT04418765) and EudraCT (2019–004497-25).

### Patients

Adults 18–75 years of age (inclusive) who had an onset of migraine (ICHD-3) at or before 50 years of age; a history of chronic or episodic migraine (ICHD-3 [19]) for ≥12 months before screening; and documented evidence of treatment failure with 2 to 4 preventive migraine medications in the past 10 years (due to inadequate efficacy, safety and/or tolerability reasons, or contraindications) were considered for study participation. However, they were not permitted to enroll if they had documented evidence of prior unsuccessful preventive treatment with an agent targeting the CGRP pathway; history or diagnosis of other headache types; history of clinically significant cardiovascular disease; use of beta-blockers, anticonvulsants, tricyclics, calcium channel blockers, angiotensin II receptor antagonists, or other medications locally approved for prevention of migraine within 1 week prior to the screening visit; use of oral anti-CGRPs for acute treatment < 4 weeks prior to the screening visit; use of eptinezumab or other monoclonal antibody targeting the CGRP pathway < 24 weeks prior to the screening visit; use of CNS- and migraine-related devices or injectable therapy < 8 weeks prior to the screening visit; use of botulinum toxin for migraine or any other medical/cosmetic reason in the head and/or neck region < 16 weeks prior to the screening visit; or use of monoamine oxidase inhibitors, ketamine, methysergide, methylergonovine, or nimesulide < 12 weeks prior to the screening visit.

Patients with concurrent diagnosis of medication-overuse headache (MOH) were permitted (confirmed at screening using ICHD-3 [19]), as were patients using stable doses (for ≥12 weeks prior to screening) of acute headache medications.

### Assessment of work productivity

The Work Productivity and Activity Impairment measure specific to migraine (WPAI:M; Additional file 1: Supplement 1) was used to evaluate the impact of migraine on each patient’s ability to work and participate in regular daily activities [20, 21]. The WPAI:M is a self-rated tool that comprises 6 questions addressing: current employment (Q1; yes or no); number of hours missed due to migraine during the preceding 7 days (Q2); number of hours missed due to other reasons during the preceding 7 days (Q3); number of hours worked during the preceding 7 days (Q4); the extent to which migraine affected productivity while at work during the preceding 7 days (Q5; 11-point visual analog scale); and the extent to which migraine affected the ability to do regular daily activities during the preceding 7 days (Q6; 11-point visual analog scale). Importantly, only survey respondents who were employed were asked to complete Q2 to Q5. Four subscores were calculated based on raw score responses: absenteeism, or the percent work time missed due to migraine, calculated as 100*Q2/[Q2 + Q4]; presenteeism, or the percent impairment while working due to migraine, calculated as 100*Q5/10; work productivity loss, or the percent overall work impairment due to migraine, calculated as

$$100\left[\frac{Q2}{Q2+Q4}+\left(1-\frac{Q2}{Q2+Q4}\right)\left(\frac{Q5}{10}\right)\right]$$

and activity impairment, or the percent activity impairment due to migraine, calculated as 100*Q6/10. Higher scores were indicative of greater impact/impairment. Raw scores captured by the WPAI measure are reported in Additional file 2: Supplement 2, Table S1.

The WPAI:M was administered every 4 weeks beginning with the baseline site visit. It was completed prior to infusion at the baseline, Week 12, and Week 24 site visits. For post-baseline site visits, the WPAI:M could be completed at the site or in the remote setting < 3 days prior to the scheduled site visit. WPAI:M assessments were scheduled to be done by telephone contact during non–site-visit weeks and had to be completed in the remote setting on the day or < 3 days prior to the scheduled phone contact.

### Statistical analyses

As an exploratory endpoint, no sample size calculations were made for the WPAI:M specifically. In the DELIVER study, based on simulations, the power was calculated to be at least 90% for the primary endpoints and at least 68% for individual key secondary endpoints [17].

The WPAI:M analysis included data from all patients who received at least 1 dose of study medication and had at least 1 valid post-baseline 4-week assessment of monthly migraine days (MMDs) in Weeks 1–12. Summary statistics (n, arithmetic mean, SD, median, lower and upper quartiles, minimum and maximum values) were calculated for continuous variables and counts/percentages for categorical variables. Denominators for each variable were based on the number of patients with non-missing values at a given visit or during the assessment period. A restricted maximum likelihood (REML)-based mixed model for repeated measures (MMRM) was used to analyze changes from baseline in WPAI:M questionnaire subscores (absenteeism, presenteeism, work productivity loss, and activity impairment). The model included the following fixed effects: visit, country, stratification factor (MHDs at baseline: ≤14/> 14) and treatment as factors, baseline WPAI:M subscores as a continuous covariate, baseline score-by-visit interaction, treatment-by-visit interaction, and stratum-by-visit interaction. Correlations between WPAI domain subscores (at Week 12) and additional endpoints that measure disease status were calculated using Spearman and Pearson correlation coefficients. A subgroup analysis was conducted in patients stratified by episodic (EM; ≤14 monthly headache days) or chronic migraine (CM; ≥15 monthly headache days for > 3 months) [19].

## Results

The assessment population included 890 individuals who received at least 1 dose of study drug, had a valid baseline assessment, and had at least 1 valid post-baseline 4-week assessment of MMDs in Weeks 1–12, which included 299 patients who received eptinezumab 100 mg, 293 who received eptinezumab 300 mg, and 298 who received placebo. In total, 845 of the 890 patients comprising the full analysis set had a valid baseline WPAI:M score for the activity impairment subcategory, but 842 patients were included in the post-baseline analyses due to incomplete data collection. A total of 609 out of 890 patients had valid presenteeism scores and work productivity loss scores at baseline, with 596 patients included in the change-from-baseline analyses. For absenteeism, a total of 623 patients had valid baseline scores and 605 patients were included in the changes-from-baseline analyses. Patients were predominantly female (*n* = 800/890 [90%]) and the mean age of study participants was 43.8 years. A total of 110/890 (12.4%) had an MOH diagnosis. Most participants (*n* = 550/890 [61.8%]) experienced 2 prior treatment failures, while 277/890 patients (31.1%) experienced 3 prior failures and 60/890 patients (6.7%) experienced 4 prior failures. Lack of efficacy (99.9%) and safety/tolerability (55.5%) were the most prevalent causes of treatment failure within the assessment population of DELIVER.

### Effects on work productivity (WPAI:M)

Raw responses to each WPAI:M question at baseline, week 4, and week 12 are reported in Additional file 2: Supplement 2, Table S1. At baseline, 75.2%, 78.2%, and 79.4% of patients in the eptinezumab 100-mg, 300-mg, and placebo groups, respectively, indicated they were currently employed; these proportions were consistent through week 12. In those who were employed, hours worked in the preceding 7 days increased from 31.8 (baseline) to 36.2 (week 12) with eptinezumab 100 mg and from 31.4 (baseline) to 35.7 (week 12) with eptinezumab 300 mg compared to no change with placebo (baseline, 32.3 hours; week 12, 32.3 hours). Employed patients treated with eptinezumab showed numerically larger decreases in the effect of migraine on work productivity compared with placebo; scores decreased from 5.1 (baseline) to 3.2 (week 12) with eptinezumab 100 mg, 5.3 (baseline) to 2.6 (week 12) with eptinezumab 300 mg, and from 5.2 (baseline) to 4.0 (week 12) with placebo. In the total assessment population, the mean score for effect of migraine on regular daily activities was 5.9 across treatment groups; at week 12, mean scores decreased to 3.5 (100 mg) and 3.2 (300 mg) with eptinezumab compared with 4.5 with placebo.

**Fig. 1:**
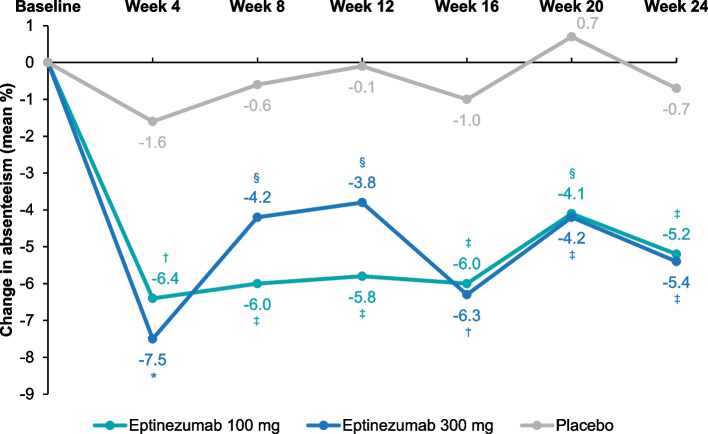
Mean Changes From Baseline in WPAI:M Absenteeism Subscores. Mean changes from baseline were calculated using LSMeans from a mixed model for repeated measures. The model included the following fixed effects: visit, country, stratification factor (monthly headache days at baseline: ≤14/> 14) and treatment as factors, baseline WPAI:M absenteeism subscore as a continuous covariate, baseline score-by-visit interaction, treatment-by-visit interaction, and stratum by-visit interaction. *P < 0.0001 vs placebo; ^†^*P* < 0.001 vs placebo; ^‡^*P* < 0.01 vs placebo; ^§^*P* < 0.05 vs placebo. WPAI:M, Work Productivity and Activity Impairment: Migraine

**Fig. 2:**
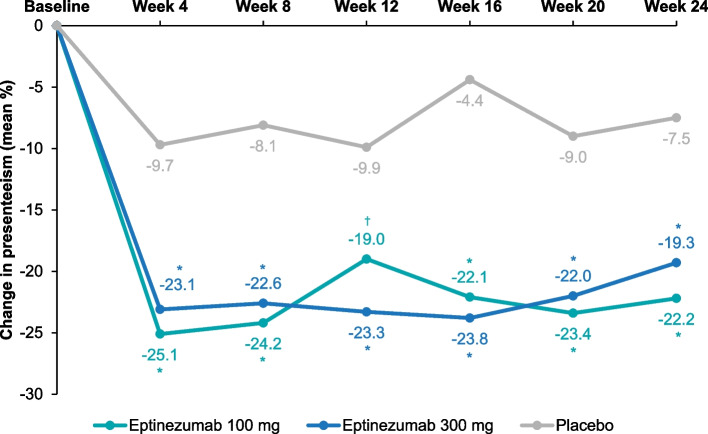
Mean Changes From Baseline in WPAI:M Presenteeism Subscores. Mean changes from baseline were calculated using LSMeans from a mixed model for repeated measures. The model included the following fixed effects: visit, country, stratification factor (monthly headache days at baseline: ≤14/> 14) and treatment as factors, baseline WPAI:M presenteeism subscore as a continuous covariate, baseline score-by-visit interaction, treatment-by-visit interaction, and stratum by-visit interaction. *P < 0.0001 vs placebo; ^†^*P* < 0.001 vs placebo. WPAI:M, Work Productivity and Activity Impairment: Migraine

Mean WPAI:M absenteeism and presenteeism subscores at baseline indicated that study participants missed approximately 12% of working hours and worked with migraine for approximately 52% of working hours during the 7 days preceding treatment, based on patients’ self-reported accounts. Eptinezumab improved absenteeism and presenteeism subscores throughout the 24-week double-blind treatment period (Table 1). The greatest change in absenteeism and presenteeism rates occurred between baseline and Week 4, after which the rates plateaued from Week 4 to Week 24. For both absenteeism and presenteeism, improvements from baseline at all timepoints were greater than those observed in the placebo group (*P* < 0.05) (Figs. 1 and 2). Subgroup analysis of WPAI:M subscores revealed that for absenteeism, there was a greater mean change from baseline in both the eptinezumab treatment groups than with placebo for both EM and CM, and all treatment groups had larger mean change from baseline for patients with CM than for patients with EM (Additional file 2: Supplement 2, Fig. S1). The mean change from baseline in presenteeism was greater in both eptinezumab treatment groups than for placebo, and the levels of change were similar between patients with EM and patients with CM (Additional file 2: Supplement 2, Fig. S2).

**Table 1:** Mean (SD) WPAI:M Subscores, by Treatment and Week

	Baseline	Week 4	Week 8	Week 12	Week 16	Week 20	Week 24
Absenteeism							
Eptinezumab 100 mg	11.4 (19.4)	4.7 (12.4)	4.7 (11.2)	5.8 (12.3)	5.7 (13.3)	6.4 (16.1)	6.0 (15.5)
Eptinezumab 300 mg	12.0 (19.3)	3.2 (8.2)	6.3 (17.4)	6.0 (16.9)	4.4 (12.9)	6.1 (15.0)	5.2 (12.6)
Placebo	12.9 (20.1)	9.5 (18.4)	10.0 (20.3)	11.3 (21.2)	9.0 (17.2)	12.2 (22.4)	9.8 (18.7)
Presenteeism							
Eptinezumab 100 mg	50.8 (25.6)	26.7 (26.6)	26.5 (26.7)	32.3 (28.1)	30.4 (28.2)	27.3 (26.2)	28.2 (25.1)
Eptinezumab 300 mg	53.4 (24.0)	26.3 (25.4)	28.0 (26.4)	26.1 (24.5)	25.3 (25.3)	26.7 (27.1)	30.4 (27.1)
Placebo	51.7 (24.2)	40.0 (25.6)	41.7 (27.5)	39.9 (26.2)	42.8 (27.4)	40.2 (29.3)	41.5 (28.3)
Work Productivity Loss							
Eptinezumab 100 mg	53.7 (26.2)	28.8 (28.0)	28.8 (28.4)	34.6 (29.6)	32.5 (29.9)	29.5 (27.9)	30.5 (27.0)
Eptinezumab 300 mg	57.0 (24.1)	27.7 (26.5)	30.2 (27.9)	28.1 (26.0)	26.9 (26.5)	29.1 (28.9)	32.5 (28.6)
Placebo	55.6 (24.7)	43.5 (27.0)	44.6 (28.9)	43.3 (27.7)	45.9 (28.8)	43.5 (31.0)	45.0 (29.4)
Activity Impairment							
Eptinezumab 100 mg	58.5 (23.5)	34.2 (28.4)	32.7 (28.6)	34.9 (27.1)	31.4 (27.5)	31.5 (27.2)	31.0 (25.9)
Eptinezumab 300 mg	59.1 (23.4)	32.6 (28.3)	31.6 (28.2)	31.9 (26.7)	29.4 (27.0)	30.5 (28.2)	33.1 (27.0)
Placebo	58.7 (23.5)	45.5 (25.4)	44.6 (26.9)	44.7 (26.9)	45.5 (26.3)	43.4 (29.0)	45.2 (27.0)

Subscores represent raw mean percentage of time. *SD* standard deviation, *WPAI:M* Work Productivity and Activity Impairment: Migraine

**Fig. 3:**
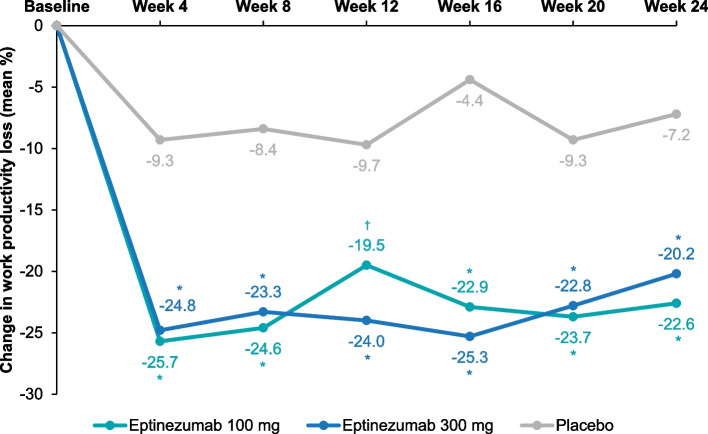
Mean Changes From Baseline in WPAI:M Work Productivity Loss Subscores. Mean changes from baseline were calculated using LSMeans from a mixed model for repeated measures. The model included the following fixed effects: visit, country, stratification factor (monthly headache days at baseline: ≤14/> 14) and treatment as factors, baseline WPAI:M work productivity loss subscore as a continuous covariate, baseline score-by-visit interaction, treatment-by-visit interaction, and stratum by-visit interaction. *P < 0.0001 vs placebo; ^†^*P* < 0.001 vs placebo. WPAI:M, Work Productivity and Activity Impairment: Migraine

**Fig. 4:**
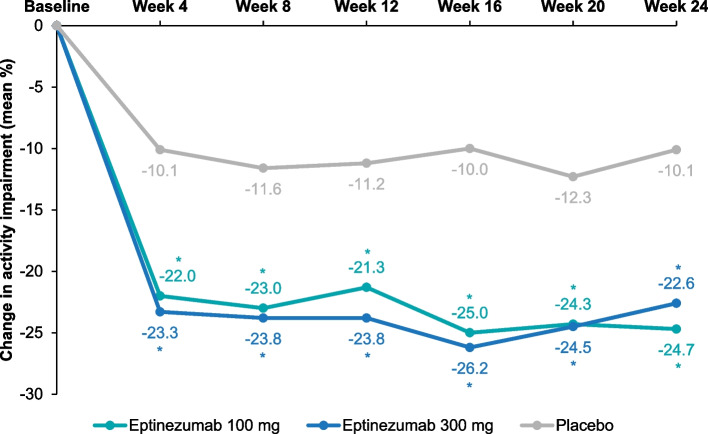
Mean Changes From Baseline in WPAI:M Activity Impairment Subscores. Mean changes from baseline were calculated using LSMeans from a mixed model for repeated measures. The model included the following fixed effects: visit, country, stratification factor (monthly headache days at baseline: ≤14/> 14) and treatment as factors, baseline WPAI:M activity impairment subscore as a continuous covariate, baseline score-by-visit interaction, treatment-by-visit interaction, and stratum by-visit interaction. *P < 0.0001 vs placebo. WPAI:M, Work Productivity and Activity Impairment: Migraine

Baseline work productivity loss scores averaged 55.4% and baseline activity impairment scores averaged 58.7%, indicating an impact of migraine attacks on work productivity and ability to complete normal daily activities [22]. Scores range from 0% to 100% after conversion, and higher scores indicate increasing impairment and decreasing productivity [2, 22]. (For comparison, patients with low monthly headache frequency [0–7 headache days/month] have been shown to have 32.1% total work productivity impairment and 30.9% total activity impairment at baseline [23].) Eptinezumab reduced work productivity loss and activity impairment subscores more than placebo throughout the double-blind treatment period (Table 1), with changes from baseline being greater in the eptinezumab treatment groups than in the group receiving placebo at all timepoints (*P* < 0.001) (Figs. 3 and 4). The greatest change in work productivity loss scores and activity impairment scores occurred between baseline and Week 4, after which the scores plateaued from Week 4 to Week 24. Subgroup analysis of WPAI:M subscores in patients with EM and CM showed that the mean change from baseline in work productivity loss subscores were greater in both eptinezumab treatment groups than with placebo and that the levels of change per treatment were similar between patients with EM and patients with CM (Additional file 2: Supplement 2, Fig. S3). For the activity impairment subscores for both EM and CM patients the mean change from baseline was greater with both eptinezumab treatment groups than with placebo, while the change from baseline for all treatments were slightly larger for EM than for CM patients (Additional file 2: Supplement 2, Fig. S4).

WPAI:M domain scores were weakly or moderately correlated with other endpoints indicative of improved disease status (Additional file 2: Supplement 2, Table S2). Spearman correlation coefficients suggested that reductions in presenteeism and work productivity loss were moderately correlated (r = 0.45 to 0.48) with reductions in MMDs and patient-identified most bothersome symptom (PI-MBS) change scores; that reductions in absenteeism were weakly correlated (r = 0.27 to 0.29) with those measures; and that reductions in activity impairment were moderately correlated (r = 0.48) with PI-MBS change scores and weakly correlated (r = 0.39) with reductions in MMDs. Weak correlation coefficients were found between all WPAI:M domains and change from baseline in the percentage of migraine attacks with severe intensity.

## Discussion

In adults with migraine and multiple prior preventive treatment failures, eptinezumab 100 mg and 300 mg administered via IV infusion every 12 weeks led to improved self-reported work productivity and reduced activity impairment compared to placebo. Benefits were observed as early as Week 4, the first post-baseline assessment. This early response is consistent with the early (within the first 4 weeks) onset of improvements in MMDs [17, 24], well-being (EQ-5D-5L) [25], symptoms (PI-MBS) [25–27], headache impact (6-item Headache Impact Test [HIT-6]) [10, 17, 25], and disease status (Patient Global Impression of Change [PGIC]) [10, 25] previously reported. Changes from baseline in MMDs in the DELIVER study showed that changes were largest during the first 4 weeks of treatment, with some patients further responding after a second dose (Weeks 13–24) [17].

Throughout its clinical development, eptinezumab has consistently demonstrated early and sustained reductions in headache/migraine frequency [10–15, 28–30] and severity [31], both characteristics of which can contribute to reduced work productivity. Results of the large National Health and Wellness Survey (NHWS) provide insight into the subjectively assessed benefit of reduced frequency on productivity. In the US NHWS (2016), each incremental increase in monthly headache-free days (0–10, 11–20, 21–26) was associated with a 5% reduction in work days missed and days of household activities missed [32]. In the European NHWS (2017), even an increase of a single headache-free day was associated with reduced absenteeism (− 3.9%), presenteeism (− 2.1%), total work impairment (− 2.1%), and activity impairment (− 1.8%); an increase of 5 headache-free days was associated with greater improvements (− 18.2%, − 10.3%, − 10.1%, and − 8.7%, respectively) [33].

The analysis population of DELIVER consisted of patients for whom migraine severity was a key factor, as the study included patients with 2–4 prior preventive treatment failures at baseline. Eptinezumab treatment previously led to sustained and consistent reductions in PI-MBS severity in the PROMISE-2 study in chronic migraine, with 45–66% of eptinezumab-treated patients reporting much or very much improvement in their PI-MBS as early as Week 4 relative to placebo recipients (29–41%) [26]. In DELIVER, 50–56% of eptinezumab-treated patients reported much or very much improvement in their PI-MBS at the Week 12 and Week 24 time points, compared with 19–23% of patients receiving placebo [27]. Additionally, a higher percentage of the patients in the placebo group of the PROMISE-2 study were responsive to treatment (~ 30% and higher) [26] compared to patients in the placebo group of DELIVER (~ 20% and higher) [25]. The differences in placebo response between these two populations may be indicative of the patients’ lowering expectations for treatment success as the number of prior treatment failures increases, providing further context for the improvements in scores reported in this work. Finally, severity as scored by the PI-MBS measure was found to correlate strongly with PGIC scores, the latter of which measured patients’ overall quality of life [26]. Interestingly, correlation coefficients in the current study suggest that improvements for all domains were most strongly correlated with changes in PI-MBS, closely followed by changes in MMDs and both more than changes in migraine attacks with severe intensity, underscoring the need to address additional migraine symptoms beyond headache pain.

The current findings of improved WPAI scores after eptinezumab treatment are consistent with those reported in studies of other CGRP inhibitors but were numerically greater in magnitude than the other studies. In LIBERTY, the change from baseline in WPAI scores relative to placebo varied from − 2.6 to − 13.1 (*P* < 0.05) after treatment with erenumab 140 mg, reflecting improvements in 3 of the 4 WPAI subscores [34]. In FOCUS, fremanezumab administered quarterly or monthly improved all 4 WPAI subscores, with changes from baseline ranging from − 4.7 to − 20.0, during the last 4 weeks of the 12-week double-blind treatment period (all *P* < 0.05 vs placebo except absenteeism with quarterly fremanezumab) [35]. Lastly, in the HALO-CM study, quarterly or monthly fremanezumab improved all 4 WPAI subscores during the last 4 weeks of the 12-week double-blind treatment period, with changes from baseline ranging from − 12.9 to − 16.6 (all except absenteeism with both regimens and activity impairment with monthly fremanezumab being greater than changes in the placebo group [*P* < 0.05]) [36]. In all of these studies, the benefit of CGRP inhibition on presenteeism was of greater magnitude than the benefit on absenteeism. This likely reflects the fact that many patients with migraine continue to work while experiencing symptoms [3, 5].

### Limitations

The duration of the placebo-controlled portion of DELIVER was 24 weeks, with not all patients completing the WPAI:M at baseline or every prespecified endpoint. WPAI:M was not included in the testing hierarchy and the *P*-values reported here have not been controlled for multiplicity. While changes in WPAI:M subscores during this time period suggest an early onset and short-term maintenance of beneficial effects on work productivity and activity, results from longer-term evaluations are needed to assess the extent of the durability of this positive response. Future work may provide insight on the change from baseline in WPAI scores over weeks 37–72. Additionally, while there were moderate correlations between MMDs and changes in WPAI:M scores, the analysis is not comprehensive and factors beyond MMDs are likely to impact scores. The DELIVER population was not diverse. Patients were predominantly female (89.9%) and white (96.0%), and most patients had two prior preventive treatment failures (61.8%). Thus, the findings reported here may not be generalizable to the broader migraine population, but subjects did hold a variety of occupational roles, including salesperson, manager, administrator, working professional, and skilled laborer. As a self-reported measure, the WPAI:M captures patients’ perceptions of improvements made and does not objectively measure changes in absenteeism or presenteeism. While the results may or may not reflect changes in actual job performance, changes in WPAI scores were found to be stable measurements, as evidenced by the plateauing of scores from Weeks 4–12.

### Conclusion

In adults with migraine and prior preventive treatment failure, eptinezumab 100 mg and 300 mg IV every 12 weeks improved absenteeism and presenteeism and decreased work productivity loss and activity impairment more than placebo, as captured by the WPAI:M. These results indicate that eptinezumab treatment, and anti-CGRP therapies in general, can improve patient work productivity and ultimately quality of life. Reductions in migraine frequency and severity were observed early on (4 weeks post-baseline) in eptinezumab-treated patients in the DELIVER clinical trial and were sustained throughout the placebo-controlled portion of the trial.

## Supplementary Information

**Additional file 1.** Supplement 1

**Additional file 2.** Supplement 2

## Data Availability

In accordance with EFPIA’s and PhRMA’s “Principles for Responsible Clinical Trial Data Sharing” guidelines, Lundbeck is committed to responsible sharing of clinical trial data in a manner that is consistent with safeguarding the privacy of patients, respecting the integrity of national regulatory systems, and protecting the intellectual property of the sponsor. The protection of intellectual property ensures continued research and innovation in the pharmaceutical industry. Deidentified data are available to those whose request has been reviewed and approved through an application submitted to https://www.lundbeck.com/global/our-science/clinical-data-sharing.
