# Supplementary material for: Effects of eptinezumab on self-reported work productivity in adults with migraine and prior preventive treatment failure in the randomized, double-blind, placebo-controlled DELIVER study
Source: J Headache Pain. 2022 Dec 2;23(1):153. doi: 10.1186/s10194-022-01521-w (PMC9716694; doi:10.1186/s10194-022-01521-w)
Supplement: Supplementary file 1 — Additional file 1. Supplement 1 [file 10194_2022_1521_MOESM1_ESM.docx]

**Supplement 1**

**Work Productivity and Activity Impairment Questionnaire:**

**Migraine V2.0 (WPAI:MIGRAINE)** [20]

The following questions ask about the effect of your MIGRAINE on your ability to work and perform regular activities. *Please fill in the blanks or circle a number, as indicated.*

1. Are you currently employed (working for pay)? _____ NO _____ YES
    *If NO, check “NO” and skip to question 6.*

The next questions are about the **past seven days**, not including today.

1. During the past seven days, how many hours did you miss from work because of problems associated with your MIGRAINE? *Include hours you missed on sick days, times you went in late, left early, etc., because of your MIGRAINE. Do not include time you missed to participate in this study.*

_____ HOURS

1. During the past seven days, how many hours did you miss from work because of any other reason, such as vacation, holidays, time off to participate in this study?

_____ HOURS

1. During the past seven days, how many hours did you actually work?

_____ HOURS *(If “0,” skip to question 6.)*

1. During the past seven days, how much did your MIGRAINE affect your productivity while you were working?

*Think about days you were limited in the amount or kind of work you could do, days you accomplished less than you would like, or days you could not do your work as carefully as usual. If MIGRAINE affected your work only a little, choose a low number. Choose a high number if MIGRAINE affected your work a great deal.*

Consider only how much MIGRAINE affected
productivity while you were working.

| MIGRAINE had no effect on my work |  |  |  |  |  |  |  |  |  |  |  | MIGRAINE completely prevented me from working |
| --- | --- | --- | --- | --- | --- | --- | --- | --- | --- | --- | --- | --- |
|  | 0 | 1 | 2 | 3 | 4 | 5 | 6 | 7 | 8 | 9 | 10 |  |

CIRCLE A NUMBER

1. During the past seven days, how much did your MIGRAINE affect your ability to do your regular daily activities, other than work at a job?

*By regular activities, we mean the usual activities you do, such as work around the house, shopping, childcare, exercising, studying, etc. Think about times you were limited in the amount or kind of activities you could do and times you accomplished less than you would like. If MIGRAINE affected your activities only a little, choose a low number. Choose a high number if MIGRAINE affected your activities a great deal.*

Consider only how much MIGRAINE affected your ability
to do your regular daily activities, other than work at a job.

| MIGRAINE had no effect on my daily activities |  |  |  |  |  |  |  |  |  |  |  | MIGRAINE completely prevented me from doing my daily activities |
| --- | --- | --- | --- | --- | --- | --- | --- | --- | --- | --- | --- | --- |
|  | 0 | 1 | 2 | 3 | 4 | 5 | 6 | 7 | 8 | 9 | 10 |  |

CIRCLE A NUMBER

WPAI:MIGRAINE V2.0 (US English)
