# Supplementary material for: Effects of eptinezumab on self-reported work productivity in adults with migraine and prior preventive treatment failure in the randomized, double-blind, placebo-controlled DELIVER study
Source: J Headache Pain. 2022 Dec 2;23(1):153. doi: 10.1186/s10194-022-01521-w (PMC9716694; doi:10.1186/s10194-022-01521-w)
Supplement: Supplementary file 2 — Additional file 2. Supplement 2 [file 10194_2022_1521_MOESM2_ESM.docx]

**SUPPLEMENT 2**

**Table S1.** Raw scores on the WPAI:M at baseline, week 4, and week 12

|  | **Eptinezumab  100 mg** | **Eptinezumab  300 mg** | **Placebo** |
| --- | --- | --- | --- |
| **Q1: Currently employed: Yes** |  |  |  |
| Baseline, n/N (%) | 206/274 (75.2%) | 223/285 (78.2%) | 227/286 (79.4%) |
| Week 4, n/N (%) | 212/282 (75.2%) | 213/276 (77.2%) | 224/284 (78.9%) |
| Week 12, n/N (%) | 219/291 (75.3%) | 225/288 (78.1%) | 233/297 (78.5%) |
| **Q2: Missed work hours due to migraine*** |  |  |  |
| Baseline, mean (range) N | 3.6 (0, 46) 206 | 4.0 (0, 72) 223 | 5.3 (0, 168) 227 |
| Week 4, mean (range) N | 1.8 (0, 48) 212 | 1.3 (0, 36) 213 | 3.7 (0, 48) 224 |
| Week 12, mean (range) N | 2.5 (0, 56) 219 | 2.8 (0, 62) 225 | 3.7 (0, 42) 233 |
| **Q3: Missed work hours due to other reasons*** |  |  |  |
| Baseline, mean (range) N | 3.7 (0, 48) 206 | 3.0 (0, 72) 223 | 2.9 (0, 40) 227 |
| Week 4, mean (range) N | 2.9 (0, 72) 212 | 2.5 (0, 48) 213 | 3.5 (0, 96) 224 |
| Week 12, mean (range) N | 2.9 (0, 40) 219 | 2.3 (0, 45) 225 | 3.1 (0, 40) 233 |
| **Q4: Hours actually worked*** |  |  |  |
| Baseline, mean (range) N | 31.8 (0, 96) 206 | 31.4 (0, 125) 223 | 32.3 (0, 80) 227 |
| Week 4, mean (range) N | 33.2 (0, 111) 212 | 35.8 (0, 159) 213 | 33.1 (0, 168) 224 |
| Week 12, mean (range) N | 36.2 (0, 98) 219 | 35.7 (0, 165) 225 | 32.3 (0, 90) 233 |
| **Q5: Work productivity affected by migraine*^†‡^** |  |  |  |
| Baseline, mean (SD) N | 5.1 (2.56) 191 | 5.3 (2.4) 206 | 5.2 (2.42) 212 |
| Week 4, mean (SD) N | 2.7 (2.66) 192 | 2.6 (2.54) 200 | 4.0 (2.56) 208 |
| Week 12, mean (SD) N | 3.2 (2.81) 206 | 2.6 (2.45) 206 | 4.0 (2.62) 215 |
| **Q6: Regular daily activities affected by migraine^‡^** |  |  |  |
| Baseline, mean (SD) N | 5.9 (2.35) 274 | 5.9 (2.34) 285 | 5.9 (2.35) 286 |
| Week 4, mean (SD) N | 3.4 (2.84) 282 | 3.3 (2.83) 276 | 4.6 (2.53) 284 |
| Week 12, mean (SD) N | 3.5 (2.71) 291 | 3.2 (2.67) 288 | 4.5 (2.69) 297 |

Scores on the WPAI:M individual questions presented in this table were based on observed data.

*Only patients who were currently employed (based on Q1) answered this question.

^†^Only patients who worked for >0 hours in the past 7 days (based on Q4) answered this question.

^‡^Rated on scale from 0 (no effect) to 10 (completely prevented).

SD, standard deviation; WPAI:M, Work Productivity and Activity Impairment: Migraine.

**Figure S1.** Mean Changes From Baseline in WPAI:M Absenteeism Subscores in Patients With (A) Episodic Migraine and (B) Chronic Migraine

The estimated means are from a mixed model for repeated measures with month (Weeks 1-4, Weeks 5-8, Weeks 9-12, Weeks 13-16, Weeks 17-20, Weeks 21-24), country and treatment as factors, baseline score as a continuous covariate, treatment-by-month interaction and baseline score-by-month interaction.

Post hoc p-values: **P*<0.0001 vs placebo; ^†^*P*<0.001 vs placebo; ^‡^*P*<0.01 vs placebo; ^§^*P*<0.05 vs placebo.

BL, baseline; WPAI:M, Work Productivity and Activity Impairment: Migraine.

**Figure S2**. Mean Changes From Baseline in WPAI:M Presenteeism Subscores in Patients With (A) Episodic Migraine and (B) Chronic Migraine

The estimated means are from a mixed model for repeated measures with month (Weeks 1-4, Weeks 5-8, Weeks 9-12, Weeks 13-16, Weeks 17-20, Weeks 21-24), country and treatment as factors, baseline score as a continuous covariate, treatment-by-month interaction and baseline score-by-month interaction.

Post hoc p-values: **P*<0.0001 vs placebo; ^†^*P*<0.001 vs placebo; ^‡^*P*<0.01 vs placebo; ^§^*P*<0.05 vs placebo.

BL, baseline; WPAI:M, Work Productivity and Activity Impairment: Migraine.

**Figure S3.** Mean Changes From Baseline in WPAI:M Work Productivity Loss Subscores in Patients With (A) Episodic Migraine and (B) Chronic Migraine

The estimated means are from a mixed model for repeated measures with month (Weeks 1-4, Weeks 5-8, Weeks 9-12, Weeks 13-16, Weeks 17-20, Weeks 21-24), country and treatment as factors, baseline score as a continuous covariate, treatment-by-month interaction and baseline score-by-month interaction.

Post hoc p-values: **P*<0.0001 vs placebo; ^†^*P*<0.001 vs placebo; ^‡^*P*<0.01 vs placebo; ^§^*P*<0.05 vs placebo.

BL, baseline; WPAI:M, Work Productivity and Activity Impairment: Migraine.

**Figure S4**. Mean Changes From Baseline in WPAI:M Activity Impairment Subscores in Patients With (A) Episodic Migraine and (B) Chronic Migraine

The estimated means are from a mixed model for repeated measures with month (Weeks 1-4, Weeks 5-8, Weeks 9-12, Weeks 13-16, Weeks 17-20, Weeks 21-24), country and treatment as factors, baseline score as a continuous covariate, treatment-by-month interaction and baseline score-by-month interaction.

Post hoc p-values: **P*<0.0001 vs placebo; ^†^*P*<0.001 vs placebo; ^‡^*P*<0.01 vs placebo.

BL, baseline; WPAI:M, Work Productivity and Activity Impairment: Migraine.

**Table S2.** Spearman correlations* of WPAI total domain scores with other endpoints of improved disease status.

| **WPAI:M domain** | **PI-MBS score**^†^ **(Week 12)** | **Change from baseline in MMDs (Weeks 9-12)** | **Change from baseline in % of migraine attacks with severe intensity (Weeks 9-12)** |
| --- | --- | --- | --- |
| **Absenteeism** | 0.28832 (weak) | 0.26719 (weak) | 0.19205 (weak) |
| **Presenteeism** | 0.46895 (moderate) | 0.44864 (moderate) | 0.26761 (weak) |
| **Work Productivity Loss** | 0.47528 (moderate) | 0.46227 (moderate) | 0.29360 (weak) |
| **Activity Impairment** | 0.47669 (moderate) | 0.38990 (weak) | 0.26817 (weak) |

*Pearson correlation analysis produced similar results.

^†^Scores on the PI-MBS represent an amount of change.

Interpretation of correlation coefficient based on Schober et al.^1^

MMDs, monthly migraine days; PI-MBS, patient-identified most bothersome symptom; WPAI:M, Work Productivity and Activity Impairment: Migraine.

**References**

1. Schober P, Boer C, Schwarte LA (2018) Correlation coefficients: appropriate use and interpretation. Anesth Analg 126:1763-1768. doi: 10.1213/ANE.0000000000002864
